# Assessing the Relationship between Retail Store Tobacco Advertising and Local Tobacco Control Policies: A Massachusetts Case Study

**DOI:** 10.1155/2019/1823636

**Published:** 2019-09-18

**Authors:** Bukola Usidame, Edward Alan Miller, Joanna E. Cohen

**Affiliations:** ^1^Virginia Commonwealth University, Richmond, VA, USA; ^2^University of Massachusetts Boston, Boston, MA, USA; ^3^Johns Hopkins Bloomberg School of Public Health, Baltimore, MD, USA

## Abstract

**Objective:**

This study documents the extent of tobacco ads in retail stores and evaluates its association with the comprehensiveness of local tobacco control policies in the state of Massachusetts, US.

**Methods:**

Using a two-stage cluster sampling method, we sampled 419 retail stores across 42 municipalities to assess the presence and count of nine mutually exclusive tobacco ad categories. Tobacco ads by store type and municipality were analyzed using summary statistics and contingency tables. Regression models tested the association between the extent of tobacco ads and local tobacco control policy comprehensiveness.

**Results:**

Overall, 86.6% (*n* = 363) of all the retail stores had tobacco ads. On average, there were 6.7 ads per retail store (SD = 6.61) and 2804 ads across all the retail stores (range = 0 : 32). Retail stores had an average of three different categories of tobacco ads (mean = 2.98, SD = 1.84). Across all retail stores, the most frequent ad categories were power walls (80.0%) and e-cigarette ads (55.8%). Retail stores in municipalities with more comprehensive local tobacco control policies were more likely to have fewer tobacco ads (IRR = 0.92, *p* < 0.01) and a lower number of tobacco ad categories (OR = 0.88, *p* < 0.05).

**Conclusion:**

Municipalities can adopt more comprehensive tobacco control policies to help limit the extent of tobacco retail advertising. This can ultimately reduce smoking in their jurisdiction.

## 1. Introduction

Tobacco advertising, particularly at the point of sale, is used to portray tobacco products in a positive light [1–3]. In 2012, US tobacco companies spent 85.1% of advertising and promotional expenditures ($7.82 billion) on price discounts that made cigarette prices more affordable and appealing to youth and low-income customers [4]. The 2012 US Surgeon General Report concluded that tobacco advertising and promotion are responsible for the initiation and progression of tobacco use among youth [5]. In addition to encouraging impulse purchases and smoking uptake, this tobacco advertising at point of sale (POS) also discourages smoking cessation [6, 7]. In Massachusetts, tobacco company marketing is estimated to be over $121.3 million annually, primarily through promotional allowances to retail stores to push the sale of cigarettes through incentives, coupons, and prime product placement [8].

In 1998, the Massachusetts Department of Public Health conducted a statewide campaign, Operation Storefront, to document the extent of tobacco advertising in retail stores. Results showed that tobacco was the most advertised product visible to youth from outside retail stores. Each store had an average of five storefront ads that were visible from outside the retail store [9]. In 2002, Massachusetts along with 45 other states, the District of Columbia, five US territories, and four tobacco companies signed the Master Settlement Agreement (MSA), which imposed significant restrictions or prohibitions on tobacco advertising, promotion, and marketing activities [10]. Some of the MSA restrictions or prohibitions pertained to branded merchandise, billboard ads, transit advertising, and free tobacco product samples amongst many others [10]. Celebucki and Diskin [11] assessed the effect of the MSA on the extent of tobacco cigarette ads visible from outside the retail store. Contrary to expectations, the study discovered a significant increase in external cigarette advertising, an average of 4.7 external cigarette ads, mostly in gas stations and convenience stores [11]. A similar study discovered an increase in internal store ads following the MSA ban [12]. Seidenberg et al. [13] evaluated differences in storefront cigarette advertising in two communities in Massachusetts; there was an average of 5.8–6.6 external cigarette ads per retailer [13].

In 2009, the federal government enacted the Tobacco Control Act (TCA) which permitted state and local governments to further regulate tobacco ads in retail stores including licensing, placement, and categories of tobacco ads including power walls behind cash registers [14]. Some local governments, also known as municipalities, in Massachusetts have also been active in regulating tobacco sale and ads in retail stores. These regulations include a complete ban on the advertising and sale of tobacco products in drug stores, limits on retail licensing, and price restrictions set to strengthen the existing state minimum pricing laws [15]. Studies have assessed the extent of tobacco advertising with individual regulations, e.g., a reduction in price promotion ads offering free tobacco products following a ban on free tobacco products with a purchase [16]. Nevertheless, researchers have yet to assess whether the extent of tobacco ads in retail stores is associated with the stringency of local tobacco control policies following the 2009 Tobacco Control Act.

We conducted the first cross-sectional study of tobacco ads in Massachusetts following the wave of policies adopted by state and local governments after the 2009 Tobacco Control Act. We examined the extent and types of tobacco advertising. We also tested the association between the presence of tobacco ads, number of tobacco ad categories, and number of tobacco ads and comprehensiveness of local tobacco control policies. We hypothesized that jurisdictions with weaker tobacco control policies would have more tobacco ads and vice versa. Massachusetts has a progressive stance towards tobacco control policies relative to most other states in the US. For example, it has the 5^th^ highest tobacco excise tax and was the first state to prohibit the sale of tobacco products in pharmacies. In addition, it reports the 7^th^ lowest smoking rates in the country at 13.7%. The State and Community Tobacco Control Research Initiative (SCTC) has classified Massachusetts as one of the states that appear well-positioned to expand their efforts into the POS area. Thus, Massachusetts provides an excellent context for conducting tobacco advertising and compliance studies.

## 2. Methods

### 2.1. Sample Selection

Data were collected from 419 retail stores within selected Massachusetts' municipalities using a two-stage cluster sampling method from March to May 2017. We chose retail stores located in municipalities with varying comprehensiveness of tobacco regulations to ensure that retail stores in municipalities with and without comprehensive tobacco policies were eligible to be included in the sample.

The first stage involved selecting municipalities using probability-proportion to size (PPS); the selected municipalities were then split into seven groups according to the comprehensiveness of their tobacco regulations [17]. Massachusetts has 351 municipalities, but only municipalities with 10 or more retail stores were eligible for selection from each group; there were 182 municipalities with 10 or more retail stores. We selected 10 or more retail stores for cost and convenience purposes. The comprehensiveness of tobacco regulations was measured using the five tobacco control priority policies tracked by the Department of Public Health's Massachusetts Tobacco Control Program (MTCP). All tracked policies used in this study are listed in Section 2.3. The more tobacco control policy areas a municipality covers, the more stringent its stance on tobacco control is presumed to be. Based on these data, the MTCP grouped all the municipalities in Massachusetts according to the number of key policy areas that have been adopted. Municipalities that implemented all five policies were classified at the highest level, while those without any tobacco policies implemented were classified at the lowest level. This method produced six different groups according to whether municipalities have adopted all five, four, three, two, one, or none of the laws being tracked. Group 1 had the most comprehensive tobacco control policies; Group 6 had the least comprehensive tobacco control policies. The city of Boston was included as a separate group (Group 7) considering its size, uniqueness, and importance in Massachusetts.

**Figure 1:**
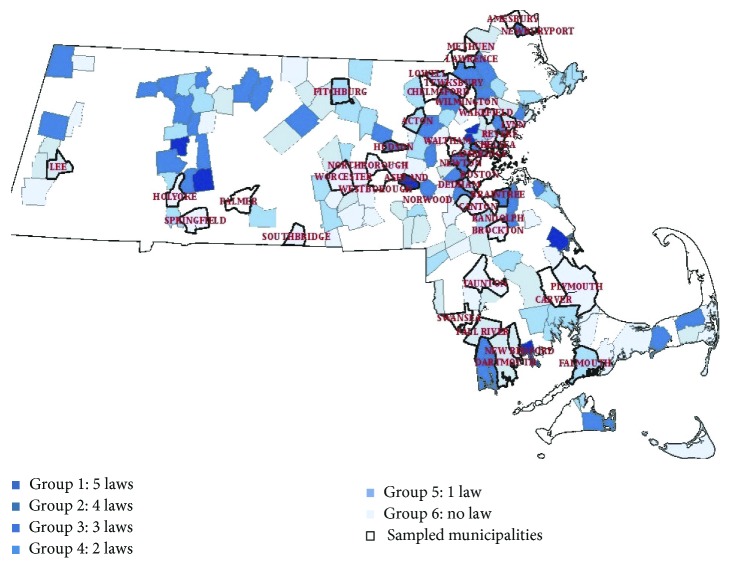
Sampled municipalities across local tobacco control policy strength.

Following municipality selection across the seven groups noted (Figure 1), an average of ten retail stores was selected per municipality using a simple random sampling method. The retail stores were selected using a comprehensive listing of tobacco retail stores, publicly available from the Massachusetts Department of Revenue's official website [15]. Information on each cigarette retailer included the business name, retail address, and license number.

### 2.2. Data Collection

Data on tobacco advertising were collected from the retail stores using a standardized observation instrument [18–20]. Store type was gathered by direct observation during the data collection process. The process resulted in a sample of 419 retail stores within 42 municipalities. We customized the TAPS observation checklist to select popular tobacco products such as cigarettes, cigars, and smokeless tobacco, as well as other advertising categories that match the provisions of local advertising regulations including flavored tobacco products, e-cigarettes, and discounts.

**Figure 2:**
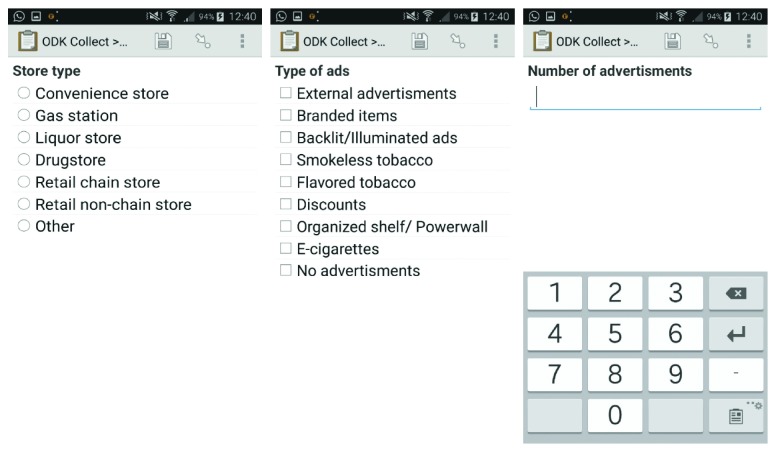
Screenshot of the survey on mobile device using Open Data Kit (ODK) Software.

Data were collected electronically by the first author using an Android data collection software called Open Data Kit (ODK) (https://opendatakit.org). The form was uploaded on a mobile device through the ODK software, making it easier to answer all the questions on the form using a phone right within each store (Figure 2). The use of ODK facilitated immediate and inconspicuous data entry. The data collector was trained to recognize tobacco advertising activities and use the observation checklist to document information relevant to the purpose of the study. The data collector spent a few minutes outside the store observing the external ads and entering the quantity and category into the phone, while seemingly browsing or sending messages on the phone. Upon entering the retail store, the data collector walked through every aisle in the store observing tobacco ads, paying primary attention to the register, waiting in line, or making a small purchase. Once each category of tobacco ad had been counted, the data were entered into the phone while in the store. Visiting the stores without notice provided an opportunity to collect data that had not been influenced by the store owners who, for example, might have altered their store displays if they knew the assessment was going to take place. Because store owners did not need to “prepare” for the store visit, reliability and validity of the data collected were stronger than if they were informed about the visit beforehand [21].

### 2.3. Measures

#### 2.3.1. Tobacco Advertisements (Dependent Variable)

Measures of tobacco ads included (i) the presence or absence of tobacco ads, (ii) the number of tobacco ads, and (iii) the number of tobacco ad categories. The presence and absence of tobacco ads were recorded as binary: 1 for presence and 0 for absence. The number of tobacco ads was a count of all the tobacco ads that were on display at the store. The number of tobacco ad categories ranged from 0 to 8 including (1) external posters, (2) internal posters, (3) e-cigarette ads, (4) smokeless tobacco ads, (5) flavored tobacco ads (excluding menthol), (6) branded items, (7) power walls, (8) ads for discounts on tobacco products such as “Buy two get one free” or “Buy one get the other half-off”, and (9) backlit ads. Each tobacco product category was recorded as binary: 1 for presence and 0 for absence. External ads were recorded as conventional cigarette or cigar ads only visible from the outside of the store, e.g., on doors, windows, walls. Internal ads included posters, decals, hanging signs, and other kinds of signage of conventional cigarettes or cigars ads visible only within the store. All ad categories were mutually exclusive, as both external and internal posters excluded ads on smokeless tobacco, discounts on tobacco products, e-cigarettes, and flavored tobacco products. The specific location (inside or outside the store) was not recorded for the other ad categories: smokeless tobacco, discounts on tobacco products, e-cigarettes, and flavored tobacco products. Nine different categories of tobacco ads were assessed, but none of the retail stores had a backlit ad, resulting in the 0 to 8 range noted.

#### 2.3.2. Comprehensiveness of Tobacco Control Policies (Independent Variable)

Local tobacco control policies were based on the number of tobacco control policy areas covered beyond tobacco advertising at POS. Data for this variable were obtained from the Massachusetts Tobacco Control Program (MTCP). Municipalities were grouped according to how many of the five key policy areas had been adopted: (1) policies that limit tobacco retail sales permits, (2) minimum pricing for cigars, (3) policies regulating the sale of e-cigarettes and nicotine delivery products to minors, (4) a ban on all flavored tobacco products, and (5) a ban on the sale of tobacco products in pharmacies. So, municipalities with no policies were coded as 0, while municipalities with all five policy areas were coded as 5. Hence, each municipality's score on this variable ranged from 0 to 5.

#### 2.3.3. Control Variables

Median household income and the percentage of minority population in a municipality were acquired from the 2009–2013 American Community Survey (ACS) 5-year estimates. Percentage minority was defined as the percentage of the residents representing the minority population (minority defined as all other races excluding non-Hispanic white residents). Store types included convenience stores (*n* = 122), gas stations (*n* = 118), liquor stores (*n* = 73), drug stores (*n* = 10), and chain retail stores, e.g., big-box retailers such as Walmart or Kroger (*n* = 49), nonchain retail stores (*n* = 16), and other store types such as tobacco shops, membership-only retail stores, fashion warehouses, bars, and private clubs (*n* = 31).

### 2.4. Analysis

Data were exported to Excel through the ODK software and transferred to Stata 15.0 for analysis. Mixed-effects logistic regression, ordinal logistic regression, and negative binomial regression were used to assess the relationship between the different tobacco ad variables and the comprehensiveness of each municipality's tobacco control local policies. Because the stores were clustered within the municipalities, all regression models were estimated using a multilevel approach. Robust standard errors were also used, which produce better variance estimates for clustered data.

## 3. Results

The sample included 419 retail stores; more than half were convenience stores (29.1%) or gas stations (28.1%) (Table 1). The remaining stores included liquor stores (17.5%), chain retail stores (11.7%), other retail store types (7.4%) such as smoke shops, private clubs, big-box stores, bars, and a fashion warehouse, nonchain retail stores (3.8%), and drug stores (2.4%). There was an average of 10 retail stores per municipality in the sample. Data were collected from five to ten retail stores from each of the municipalities and 44 stores in Boston.

### 3.1. Overall Tobacco Advertisements

Overall, 363 retail stores (86.6%) had tobacco advertising (Table 1). The remaining 56 retail stores (13.4%) had no form of tobacco advertising but sold tobacco products. There were 2804 ads spread across the 363 retail stores with ads, ranging from one to 32 ads per store. On average, there were 6.69 ads per retail store (SD = 6.61).  Table 2 reports the retail store average across the municipalities. On a municipality level, all 42 municipalities had one or more retail stores with tobacco ad(s); on average, 8.6 (SD = 4.6) stores had ads per municipality (Table 2). Each municipality had an average of 6.7 tobacco ads (SD = 2.8) per retail store (Table 2).

On average, retail stores had three different categories of tobacco ads (mean = 2.98, SD = 1.84) (Table 1). About 80% of retail stores had a power wall. Other frequent ad categories were e-cigarettes (56%) and external posters (48.6%). Ninety-one retail stores (21.7%) had at least one discount ad, and twenty-two retail stores (6.2%) had at least one flavored tobacco ad. Only 3% of retail stores had branded tobacco ads including shopping carts and welcome signs.

### 3.2. Tobacco Advertisement across Retail Store Types

Of the 2804 tobacco ads, the highest proportion was found in gas stations (*n* = 1096; 39.1%), followed by convenience stores (*n* = 1016; 36.2%), liquor stores (*n* = 309; 11.0%), chain retail stores (*n* = 187; 6.7%), other store types (*n* = 105; 3.7%), nonchain retail stores (*n* = 78; 2.8%), and drug stores (*n* = 13; 0.4%).  Table 3 presents the relationship between tobacco advertising and retail store type. Gas stations and convenience stores had the highest proportion of tobacco ads in most tobacco ad categories. Over half of gas stations (65.3%) and convenience stores (59.0%) had external ads compared to just 28.6% of chain retail stores (*p* < 0.001). Similarly, gas stations (61.0%, 92.4%) and convenience stores (51.6%, 82.8%) were more likely to have smokeless tobacco ads and power walls compared to 19.4% and 51.6% of other store types, respectively (*p* < 0.001, *p* < 0.001). Whereas gas stations (78%) and convenience stores (60.7%) also had the highest proportions of e-cigarette ads and drug stores (30%) and other stores (32.3%) had the lowest proportions (*p* < 0.001). Ads on discounts appeared most frequently in convenience stores (36.1%) and nonchain retail store types (25%), followed by gas stations (19.5%) and liquor stores (12.3%), and the other store categories examined (<25%) (*p* < 0.001). Internal posters were most common in convenience stores (40.7%) and least common in other store types (6.5%) (*p* < 0.001). No retail store had backlist ads; the few branded items were found in convenience stores (5.7%) and gas stations (0.3%) (*p* < 0.427). Flavored tobacco ads were relatively uncommon as well, appearing in small proportions of convenience stores (9.0%), gas stations (5.9%), liquor stores (9.6%), and other store types (3.2%) only (*p* < 0.201).

### 3.3. Tobacco Advertisements and Other Variables

Results from the mixed-effects logistic and negative binomial regression models are presented in Table 4 After controlling for percentage minority population, income, and store type, the odds of tobacco being present in stores was 16% lower for retail stores in municipalities with more comprehensive local tobacco control policies (OR = 0.84, *p* < 0.01). Also, there was a significant negative relationship between the presence of a flavored tobacco ad and a ban on flavored tobacco products (OR = 0.05, *p* < 0.01). However, the association between the ban on discounts for tobacco products and the advertising of discounts in retails stores was not statistically significant.

The odds of a retail store having seven tobacco ad categories, the highest number of ad categories (Table 1) compared to having six or fewer tobacco ad categories, was 12% lower for retail stores in municipalities with more comprehensive local tobacco control policies relative to other retail stores (OR = 0.88, *p* < 0.05). The odds of a retail store having an additional tobacco ad was 9% lower for retail stores in municipalities with more comprehensive local tobacco control policies compared to others (IRR = 0.91, *p* < 0.001). The tobacco ad variables were also associated with different store types. The odds of having a tobacco ad, the number of tobacco ad categories, or an additional tobacco ad was significantly associated with the retail store type. The odds of having a tobacco ad in a gas station was 5.5 times higher relative to a convenience store (OR = 5.49, *p* < 0.05).

On the other hand, the estimated rate of having an additional tobacco ad in a chain retail/liquor store or a nonchain retail/drug store/other store type was 54% and 51% lower (IRR = 0.46, *p* < 0.001; IRR = 0.49, *p* < 0.001) compared to convenience stores. Similarly, the odds of a retail store having seven tobacco ad categories compared to having six or fewer tobacco ad categories in a chain retail/liquor store (OR = 0.19, *p* < 0.001) or a nonchain retail/drug store/other store type (OR = 0.22, *p* < 0.001) was 81% and 78% lower, respectively, compared to convenience stores. All other retail store type associations were insignificant.

## 4. Discussion

This study reveals that the sampled retail stores in Massachusetts had an average of seven tobacco ads and three tobacco ad categories. Like previous studies, convenience stores and gas stations had most of the tobacco ads while drug stores and nonchain retail stores had the least tobacco ads [22, 23]. Likewise, each sampled gas station had an average of four different categories of tobacco ads while each drug store had an average of one category of tobacco ad.

As mentioned earlier, external and internal ads in this present study, which were only cigarette and cigar ads, excluded ads on smokeless tobacco, discounts, e-cigarettes, and flavored tobacco products, which were recorded separately, thereby potentially underestimating the number of all external ads. Some of the e-cigarette ads, smokeless tobacco ads, flavored tobacco ads and ads of discounts on tobacco products may have been located at the storefront. This suggests that the range of ads visible from outside the store may range from 2.8 (external ads) to 4.4 (all tobacco ads excluding indoor ads, power walls, and branded items). This range is generally consistent with the average number of external ads reported in previous surveys pre-TCA, including 5 external cigarette, cigar, and smokeless tobacco ads in 1998 [9], 4.7 external cigarette ads reported in 2000 [11] and 6.2 external cigarette ads from two communities reported in 2010 [13]. Together these findings suggest that there has been little or no reduction in the extent of external tobacco ads pre to post-TCA. Youth or adolescents in Massachusetts continue to be exposed to an average of four to five tobacco ads via the retail storefront, which is higher than a national average of 3 tobacco ads per retail store type [24].

Notably, over half of the retail stores in the sample had e-cigarette ads. All the sampled retail store types had an average of one e-cigarette ad. While over 60% of the convenience stores and gas stations had e-cigarette ads, over 30% of the drug stores and other store types also had e-cigarette ads. The high visibility of e-cigarette ads in retail stores may signal a parallel surge with increasing use of e-cigarettes [25]. The use of retail store advertising to promote the use of e-cigarettes needs to be monitored closely and addressed to help curb the increasing prevalence of e-cigarette use among youth.

Although several municipalities in Massachusetts have adopted policies limiting or banning tobacco discounts, there is still widespread use of this advertising avenue. Approximately 22% of all the retail stores sampled had ads of discounts on tobacco products. Moreover, these categories of ads were present in each retail store type, ranging from 10% or less in drug and other retail stores to approximately 40% in convenience stores and gas stations. Discounts on tobacco products are mostly harmful to youth and adolescents who are more easily swayed by price promotions than older smokers [26]. More municipalities can completely ban the use of discounts to purchase tobacco products, closing existing advertising loopholes and strengthening the state minimum pricing law.

Smokeless tobacco was also heavily advertised, with ads appearing in 43.2% of the retail stores sampled. Smokeless tobacco manufacturers increased their advertising expenditures by about $160 million from 2014 to 2016 [27, 28]. Following the 2009 Tobacco Control Act, state and local policy officials can consider adopting tobacco advertising regulations focused on curbing exposure to smokeless tobacco ads in retail stores. About 6% of the retail stores had flavored tobacco ads, specifically convenience stores (9.0%), gas stations (5.9%), liquor stores (9.6%), and other store types (3.2%). By contrast, no flavored tobacco ads were present in the drug stores, chain stores, and nonchain stores surveyed. In 2009, the FDA banned flavored cigarettes, excluding menthol, and some municipalities in Massachusetts, like Boston, have extended the ban to all other tobacco products [29]. With the ban on flavored cigarettes, other tobacco products are beginning to push flavored versions which may, in part, explain the rising prevalence of flavored tobacco ads nationally [28]. Other municipalities can adopt policies that ban all flavored tobacco products, including menthol, to reduce the proliferation of new flavored versions of other tobacco products. Flavored tobacco products increase smoking initiation rates and make it harder for smokers to quit smoking [30]. So, a ban on flavored tobacco products should result in reduced smoking rates within the community.

Between 2013 and 2014, spending on cigarette tobacco ads inside stores increased from $55.7 million to $238.2 million [25]. Our results indicate that internal ads and power walls are still widely used across all retail store types but drug stores. Thus, people who patronize these stores, including youth and adolescents, are frequently exposed to internal ads in addition to storefront tobacco advertising. A recent study discovered that the absence of power walls reduces the risk of cigarette smoking in adolescents [31]. Internal ads and power walls are thus another policy area to consider for targeting youth smoking, including stronger enforcement and restricting tobacco advertising inside stores to adult-only locations [25].

The mixed results on the association between tobacco ads and its corresponding bans support existing studies on tobacco regulations [16, 32]. The insignificant association between the presence of price promotion ads and a ban on the use of discounts to purchase tobacco products may be because retail stores with ads of discounts on tobacco products were spread across municipalities irrespective of the presence of a corresponding ban (additional analysis not in this paper). Nevertheless, the association between the presence of price promotion ads and a ban on the use of discounts was in the expected inverse direction, though insignificant; the association may have been statistically significant if the sample size was larger. Similarly, the ban on flavored tobacco products was associated with lower odds of having flavored tobacco ads, though significant. Interestingly, additional analysis also showed that almost 95% of the retail stores with flavored tobacco ads were located in municipalities without a corresponding ban. The significant association of reduced flavored tobacco ads with a corresponding local ban may be because this policy area is already regulated on a federal level, strengthening its effectiveness [14]. In addition, these results support studies that promote effective policymaking across governments. Governments are encouraged to act as tandem institutions and not in isolation when adopting policies [33]. Following this, the federal and state governments, specifically Massachusetts, are encouraged to adopt regulations banning the use of all sorts of discounts to purchase tobacco products at the retail stores.

Also, the study supports existing studies regarding factors associated with tobacco advertisements by finding an inverse relationship between advertising (presence, number of categories, and number) and the comprehensiveness of its local tobacco policies, such as a ban on the sale of e-cigarettes to minors, ban on the sale of tobacco products in pharmacies, and a limit on tobacco permits for retail stores. Similar to the previous studies, these results suggest that more comprehensive tobacco control policies might be more effective in reducing the extent of tobacco advertising relative to weaker policies [34–36]. In addition, perhaps, to greater oversight and enforcement, the negative association between the comprehensiveness of a municipality's tobacco control policies and tobacco advertising may be attributed, in part, to the higher social unacceptability to smoking in communities with greater levels of tobacco regulation [37–39]. With more local residents opposed to pro-tobacco activities, retail store owners might be less willing to advertise tobacco products in their stores. Considering the Tobacco Control Act expanded municipalities' abilities to prohibit or restrict tobacco marketing, local governments are encouraged to implement more comprehensive bans as an avenue to limit retail store tobacco advertising.

### 4.1. Limitations

This is one of the few studies assessing the extent of tobacco retail advertising in Massachusetts and its association with local tobacco control policies. However, there are several limitations worth noting. The external and internal ads were cigarette and cigar ads only, limiting this study's ability to make comprehensive assessments of more tobacco product ads in reference to the ad location. Similarly, this study did not distinguish the location of the other tobacco ad categories that were not external ads. Apart from the power walls and branded items, the study did not indicate where the flavored tobacco ads, ads of discounts on tobacco products, smokeless tobacco ads, and e-cigarette ads were located (whether outside or inside the store). With this limitation, we were unable to make exact comparisons with the previous studies on retail store tobacco advertising. Also, we collated the regulatory provisions into an ordinal scale and as such were unable to assess the effect of each regulation.

Notably, the study is only representative of the subset of retail stores in Massachusetts municipalities with ten or more retail stores, as municipalities with less than ten retail stores were excluded from the sampling frame. Additionally, we were unable to conduct interrater reliability because there was only one data collector.

## 5. Conclusion

Despite the Surgeon General's warning that tobacco advertising and promotion cause smoking initiation and the Tobacco Control Act that allows state and local governments to regulate retail tobacco advertising, retail store tobacco advertising continues in Massachusetts. This study reveals the extent to which young people are potentially exposed to retail tobacco advertising in Massachusetts and confirms that this potential exposure is reduced in municipalities with comprehensive local tobacco control policies. In particular, tobacco companies are still channeling promotion through multiple avenues including external ads, internal ads, power walls, e-cigarettes ads, and smokeless tobacco ads. These results suggest that local policies governing advertising should be encouraged across all municipalities to limit the extent of tobacco advertising in their area, hence reducing smoking rates. Other municipalities might follow Boston's example in enacting a ban on flavored tobacco products but include a ban on menthol-flavored products as well. Also, municipalities without a ban on the sale of tobacco products with discounts could incorporate a ban to further reduce the use of discounts to sell tobacco products. Federal and state governments are also encouraged to adopt policies that are already promoted on a municipality level such as a ban on all discount types to purchase tobacco products. These findings imply that municipalities with weak tobacco control policies should strengthen them, in the expectation that less exposure to tobacco advertising is likely to reduce smoking uptake among young people [40, 41].

## Figures and Tables

**Table 1:** Descriptive statistics of all study variables across all retail stores, *n* = 419.

	# (%)/mean (SD)	Minimum	Maximum
Tobacco advertisements			
Presence of advertisements	363 (86.7%)		
Number of tobacco advertisements^a^	6.69 (6.61)	0	32
Number of tobacco advertisement categories (range)	2.89 (1.84)	0	7
Retail store type			
Convenience stores	122 (29.1%)		
Gas station	118 (28.2%)		
Liquor store	73 (17.5%)		
Drug store	10 (2.4%)		
Chain retail stores	49 (11.7%)		
Nonchain retail stores	16 (3.8%)		
Other store types^b^	31 (7.4%)		
Categories of tobacco advertisements			
Presence/number of ads (mean/SD)			
External ads	204 (48.7%)/2.8 (4.3)	0	26
Backlit ads	0 (0.0%)	0	0
Branded items	14 (3.3%)/0.03 (0.2)	0	1
Smokeless tobacco ads	181 (43.2%)/0.5 (0.8)	0	5
Flavored tobacco ads	26 (6.2%)/0.1 (0.2)	0	1
Power wall	335 (80.0%)/0.8 (0.4)	0	1
Ads of discounts on tobacco products	91 (21.7%)/0.3 (0.5)	0	6
E-cigarette ads	234 (55.8%)/0.8 (1.0)	0	8
Internal posters	125 (29.8%)/1.4 (2.8)	0	15

^a^Sum of the number of tobacco advertisements across all stores = 2,804. ^b^Other store types include tobacco shops, big-box stores, fashion stores, bars, and private clubs.

**Table 2:** Descriptive statistics of all study variables at the municipality level, *n* = 42.

	Mean (SD)	Minimum	Maximum
Tobacco advertisements			
Presence of advertisements	8.6 (4.6)	4	36
Number of tobacco advertisements^a^	6.7 (2.8)	1.6	14.5
Number of tobacco advertisement categories (range)	2.9 (0.8)	1.3	5
Retail store type			
Convenience stores	7.4 (8.3)	1	24
Gas station	4.0 (1.9)	1	8
Liquor store	2.6 (1.2)	1	5
Drug store	1.2 (0.4)	1	2
Chain retail stores	1.9 (0.7)	1	3
Nonchain retail stores	1.3 (0.4)	1	2
Other store types^b^	1.9 (1.0)	1	4
Categories of tobacco advertisements			
Presence of external ads	5.0 (3.1)	1	18
Presence of backlit ads	0 (0.0)	0	0
Presence of branded items	1.3 (0.9)	1	4
Smokeless tobacco ads	4.4 (2.1)	1	10
Presence of flavored tobacco ads	2.6 (1.4)	1	5
Presence of a power wall	8.0 (4.1)	4	32
Presence of discounts on tobacco products	3.8 (4.2)	1	20
Presence of e-cigarette ads	5.6 (2.0)	1	12
Presence of internal posters	4.0 (3.5)	1	19

*Note*. The values in this table represent the average figures for retail stores across all the sampled municipalities. ^a^Sum of the number of tobacco advertisements across all stores = 2,804. ^b^Other store types include tobacco shops, big-box stores, fashion stores, bars, and private clubs.

**Table 3:** Descriptive and bivariate statistics for tobacco advertisements categories by retail store type, *n* = 419.

	Total (*n* = 419)	Convenience (*n* = 122)	Gas station (*n* = 118)	Liquor store (*n* = 73)	Drug store (*n* = 10)	Chain retail (*n* = 49)	Nonchain (*n* = 16)	Other (*n* = 31)	*χ*2, F (df), *p* value
Presence of tobacco ads	363 (100%)	107 (87.8%)	115 (97.5%)	63 (86.3%)	8 (80.0%)	38 (77.6%)	12 (75.0%)	20 (64.5%)	21.1 (1), *p* < 0.001
Total no. of tobacco ads (count)	2804	1016	1096	309	13	187	78	105	3.7 (28), *p* < 0.001
Average no. of tobacco ads	6.7 (6.6)	8.3 (7.5)	9.3 (6.3)	4.2 (4.6)	1.3 (1.2)	3.8 (4.9)	4.9 (5.0)	3.4 (5.9)	2.2*e* + 03 (36), *p* < 0.001
Average no. of tobacco ad categories (range)	2.9 (1.8)	3.4 (2.0)	3.7 (1.3)	2.2 (1.5)	1.2 (0.9)	2.0 (1.7)	2.4 (2.0)	1.5 (1.9)	24.1 (6), *p* < 0.001
External ads	204 (100%)	72 (59.0%)	77 (65.3%)	23 (31.5%)	0 (0.0%)	14 (28.6%)	7 (43.8%)	11 (35.5%)	46.5 (6), *p* < 0.001
Backlist ads	0 (100%)	0 (0.0%)	0 (0.0%)	0 (0.0%)	0 (0.0%)	0 (0.0%)	0 (0.0%)	0 (0.0%)	
Branded items	14 (100%)	7 (5.7%)	4 (0.3%)	3 (0.04%)	0 (0.0%)	0 (0.0%)	0 (0.0%)	0 (0.0%)	6.0 (6), *p* < 0.427
Smokeless tobacco ads	181 (100%)	63 (51.6%)	72 (61.0%)	18 (0.3%)	0 (0.0%)	15 (30.6%)	7 (43.8)	6 (19.4%)	47.0 (6), *p* < 0.001
Flavored tobacco ads	26 (100%)	11 (9.0%)	7 (5.9%)	7 (9.6%)	0 (0.0%)	0 (0.0%)	0 (0.0%)	1 (3.2%)	8.5 (6), *p* < 0.201
Power wall	335 (100%)	101 (82.8/%)	109 (92.4%)	51 (69.9%)	8 (80.0%)	38 (77.6%)	12 (75.0%)	16 (51.6%)	32.6 (6), *p* < 0.001
E-cigarette ads	234 (100%)	74 (60.7%)	92 (78.0%)	27 (37.0%)	3 (30.0%)	21 (42.9%)	7 (43.8%)	10 (32.3%)	49.1 (6), *p* < 0.001
Ads of discounts	91 (100%)	49 (40.2%)	23 (19.5%))	9 (12.3%)	1 (10.0%)	3 (6.1%)	4 (25.0%)	2 (6.5%)	40.7 (6), *p* < 0.001
Internal posters	125 (100%)	44 (36.1%)	48 (40.7%)	20 (27.4%)	0 (0.0%)	9 (18.4%)	2 (12.5%)	2 (6.5%)	26.8 (6), *p* < 0.001

**Table 4:** Bivariate and multiple regression models showing the association between tobacco advertising variables and the strength of local tobacco control policies.

Variables	Presence of tobacco ads^1^		Presence of discounts		Presence of flavored tobacco ads		Range of tobacco ad categories^2^		Number of tobacco ads^3^	
	OR	AOR	OR	AOR	OR	AOR	OR	AOR	IRR	AIRR
Independent variable										
Strength of other tobacco policies	0.84+	0.84+	0.99	1.01	0.71	0.78	0.89^*∗∗∗*^	0.88^*∗*^	0.91^*∗∗∗*^	0.91^*∗∗∗*^
Ban on discounts			0.44	0.63						
Ban on flavored OTP					0.10^*∗*^	0.05^*∗∗*^				
Control variables										
Retail store level										
Store type										
Convenience stores (REF)^#^										
Gas station		5.49^*∗*^		0.76		0.54		1.22		1.23
Chain retail/liquor stores		0.50^*∗*^		0.21^*∗*^		0.37		0.19^*∗∗∗*^		0.46^*∗∗∗*^
Nonchain retail/drug store/other store types^4^		0.45		0.11^*∗∗*^		1		0.22^*∗∗∗*^		0.49^*∗∗∗*^
Municipality level										
Percent minority (% of minority population)		0.98		1.09^*∗∗*^		1.01^*∗*^		1.00		1.00
Median household income ($1,000)		1.00		1.00		1.00		1.00		1.00^*∗*^
Fit statistics										

N	419	419	419	419	419	419	419	419	419	419
AIC	330.0	311.7	369.7	345.1	164.1	161.8	1629.1	1554.5	2485.0	2421.9
BIC	342.1	344.0	385.8	381.4	180.3	193.1	1665.4	1611.0	2501.2	2458.3

^+^ *p* < 0.10 (one-tailed test). ^*∗*^*p* < 0.05, ^*∗∗*^*p* < 0.01, and ^*∗∗∗*^*p* < 0.001 (two-tailed test). ^#^Reference group. ^1^Mixed-effects logistic regression. ^2^Mixed-effects ordinal logistic regression. ^3^Mixed-effects negative binomial regression. ^4^Other store types include tobacco shops, big-box stores, fashion stores, bars, and private clubs. OR = odds ratio; AOR: adjusted odds ratio; IRR: incidence rate ratio; AIRR: adjusted incidence rate ratio.

## Data Availability

The data used to support the findings of this study are available from the corresponding author upon request.
